# Exploring the Changes in Pain Quality, Mechanical Allodynia, and Tactile Sensibility in Women With Persistent Postpartum Perineal Pain Treated With Somatosensory Rehabilitation: A Retrospective Case Series

**DOI:** 10.7759/cureus.91834

**Published:** 2025-09-08

**Authors:** Isabelle Quintal, Laurence Goulet, Joseph-Omer Dyer, Daniel Bourbonnais, Mélanie Morin

**Affiliations:** 1 Faculty of Medicine and Health Sciences, School of Rehabilitation, Université de Sherbrooke, Sherbrooke, CAN; 2 Faculty of Medicine, School of Rehabilitation, Université de Montréal, Montréal, CAN

**Keywords:** allodynia, dyspareunia, genito-pelvic pain/penetration disorder, postpartum pelvic girdle pain, somatosensory rehabilitation, tactile sensibility

## Abstract

Background

Persistent postpartum perineal pain (PPPP) is a prevalent condition characterized by provoked, unprovoked, or mixed pain. Somatosensory rehabilitation has shown significant effects in alleviating provoked pain, and, more specifically, mechanical allodynia, in other populations. Therefore, this method could be a promising approach for the treatment of PPPP. This study aims to describe changes in pain quality, mechanical allodynia, and tactile sensibility in women with PPPP treated with somatosensory rehabilitation.

Methodology

This retrospective case series describes the use of a somatosensory rehabilitation method applied to the treatment of PPPP. This intervention mainly involved tactile stimulation to treat pain, mechanical allodynia, and tactile sensibility. The study included individual outcomes of nine eligible patients treated with this method. Outcome measures included pain quality (*Questionnaire de la douleur Saint-Antoine*), surface (allodynography with a 15.0-g monofilament), and severity of mechanical allodynia (Rainbow Pain Scale), as well as tactile sensibility (pressure perception threshold, static two-point discrimination test, vibration perception threshold).

Results

All patients showed a reduction in pain quality score and resolution of mechanical allodynia in the vulva, defined by the criteria of no significant exacerbation of pain upon touch with a 15.0-g Semmes-Weinstein monofilament. Sensibility assessments revealed an improvement in tactile hypoesthesia in the area of the skin where allodynia was previously present.

Conclusions

This study shows promising results for the use of somatosensory rehabilitation in the treatment of PPPP. Further randomized controlled studies are needed to investigate its efficacy in this population.

## Introduction

Persistent pain, also referred to as chronic pain, is defined as pain lasting for over three months [1]. It is highly prevalent among postpartum women, as 61.8% to 78.8% of women report persistent postpartum perineal pain (PPPP) three months or more after childbirth. This rate is believed to vary based on the severity of perineal trauma during vaginal delivery and the time elapsed since childbirth [2]. PPPP is also associated with several other factors, including instrumental delivery, depressive symptoms, fatigue, and pelvic muscle dysfunction [3]. Recognizing this condition is crucial, as it can negatively affect sexual functioning [3,4] and quality of life [5,6].

PPPP can manifest in diverse clinical forms, categorized based on whether the pain is provoked (pain elicited by touch, such as during intercourse, tampon use, or tight clothing), unprovoked (occurring without any external simulation), or mixed (involving both unprovoked and provoked pain) [7]. Dyspareunia, or provoked pain during sexual intercourse, stands out as the predominant subtype of PPPP [3]. This provoked perineal pain can be conceptualized as a form of mechanical allodynia, defined by the International Association for the Study of Pain Taxonomy as perceived pain provoked by a touch stimulus normally perceived as painless [1,8]. Consequently, interventions targeting mechanical allodynia could potentially yield beneficial effects on provoked and even mixed PPPP.

Somatosensory rehabilitation (SR) is a standardized method that integrates both assessments and interventions for managing mechanical allodynia (i.e., provoked pain), as well as unprovoked and mixed pain [9]. In the first phase of SR intervention, the surface and severity of mechanical allodynia are assessed to monitor its evolution and identify the optimal distant area for applying tactile stimulation techniques aimed at reducing pain [9]. The first phase terminates when the mechanical concerns have been resolved. Tactile hypoesthesia in the previously affected area is usually revealed, according to studies investigating various non-genital neuropathic pain conditions and complex regional pain syndrome [10-12]. This tactile hypoesthesia is assessed to document changes over time during the second phase of SR through direct application of tactile stimulation techniques to the affected area [9,10]. Retrospective case series have reported improvements in patients treated with SR for mechanical allodynia in burn patients [13], complex regional pain syndrome [11], and various chronic pain conditions (with provoked only or mixed pain) [10]. Considering that provoked pain associated with PPPP can be likened to mechanical allodynia, SR may potentially have beneficial effects on pain experienced by women with this condition.

To date, no study has documented the effects of SR on women with PPPP. This study aimed to describe the use of SR as a novel method for both the assessment and treatment of PPPP and outline the changes in pain quality, mechanical allodynia, and tactile sensibility in women with PPPP who undergo SR intervention.

## Materials and methods

Design and setting

This retrospective case series involved women referred to undergo SR intervention for provoked or mixed PPPP. Treatment for this condition was administered at the Somatosensory Rehabilitation Center of Fribourg (Switzerland) between June 2004 and October 2018. This center is recognized as a specialized provider of SR for various chronic pain conditions. Demographic and all clinical data results (allodynic surface, allodynia severity using the rainbow pain scale, treatment duration, pressure perception threshold, static two-point discrimination test, vibration perception thresholds) were extracted by two investigators (IQ, JOD) from the electronic clinical database of this Center for all women treated for this condition during this period. The Research Ethics Committee of the University of Montréal approved this study (approval number: #16-123-CERES-D). For the purposes of this study, the ethics committee authorized us to use data collected from patients, provided that the data presented did not contain information that could identify specific individuals.

Patients

The medical records of women were eligible for inclusion in the study if they documented persistent, provoked, or mixed perineal pain. In addition, inclusion criteria required that the database documented their eligibility before starting SR, ensuring they met the following conditions: (1) between the ages of 18 to 45 (to minimize the potential influence of hormonal changes associated with menopause, and thereby excluding vulvar pain related to genitourinary syndrome of menopause); (2) perineal pain that began following childbirth and persisted at the time of enrollment; (3) reporting of provoked or mixed pain at the perineum, including the vulva and the vaginal entry, for at least three months; and (4) presence of mechanical allodynia at the perineum, defined as evoked or increased pain rated ≥3/10 on the Visual Analog Scale (VAS) upon touch with a 15.0-g (no. 5.18) Semmes-Weinstein monofilament [9]. Exclusion criteria were applied to women with documented (1) postmenopausal status; (2) neurological conditions (e.g., multiple sclerosis, stroke); (3) other urogynecological symptoms (e.g., urinary or fecal incontinence, urinary urgency); (4) pelvic organ prolapse (>1 stage on pelvic organ prolapse quantification) [14]; and (5) active urinary or vaginal infection (or within the last three months before admission to the Center). These exclusion criteria were selected to minimize clinical heterogeneity and reduce potential confounders that could interfere with the interpretation of pain-related outcomes. These exclusion criteria were also selected to minimize clinical heterogeneity and reduce confounders in pain-related outcomes. For instance, neurological conditions (e.g., multiple sclerosis, stroke) and urogynecological disorders (e.g., incontinence, pelvic organ prolapse) may independently influence pain perception. Similarly, active or recent infections were excluded to avoid transient or overlapping symptoms not related to PPPP.

Overview of assessments and interventions

**Figure 1:**
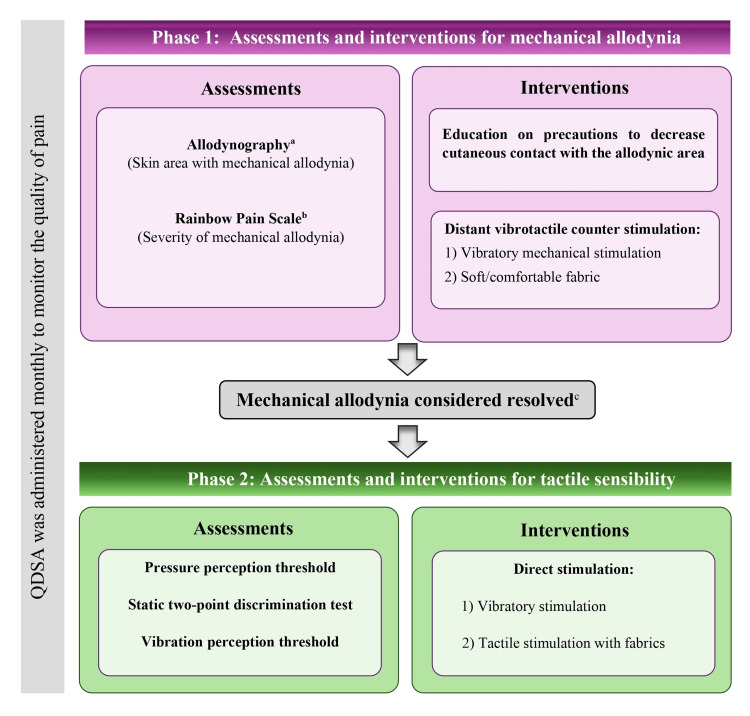
Assessments and interventions based on somatosensory rehabilitation. The left side of the figure illustrates assessments, while the right side depicts interventions for the two phases of the program. The criterion for pain exacerbation was used to determine the presence of mechanical allodynia and the transition from phase 1 to phase 2. The Questionnaire de la douleur Saint-Antoine (QDSA) was administered throughout the program to characterize pain severity. ^a^: Surface of the skin where the application of a 15-g Semmes-Weinstein monofilament provoked a pain increase of ≥10 mm on the Visual Analog Scale, reaching a minimum total of 30 mm. ^b^: Pressure pain threshold when the pain exacerbation criterion* was met using Semmes-Weinstein monofilaments. ^c^: Mechanical allodynia was considered if the application of a 15-g Semmes-Weinstein monofilament did not provoke a pain increase of ≥10 mm on the Visual Analog Scale, reaching a minimum total of 30 mm. QDSA: *Questionnaire de la douleur Saint-Antoine* Original figure created by the first author (IQ).

SR was initially developed to address persistent pain, including mechanical allodynia, unprovoked pain, or mixed pain, and to target tactile sensibility related to various non-genital conditions [9,15]. This integrated method follows a two-phase intervention, as illustrated in Figure 1. Phase 1 focuses on assessing and addressing mechanical allodynia. The assessments include allodynography, defining the skin area eliciting allodynia, and the Rainbow Pain Scale for assessing allodynia severity [16,17]. In addition, pain quality is evaluated using the *Questionnaire de la douleur Saint-Antoine* during both phases 1 and 2, with its use authorized with the kind permission of the journal *Thérapie* [18,19]. Mechanical allodynia is addressed through tactile stimulation in a territory at a distance from the allodynic area, along with precautions to decrease cutaneous contact with the allodynic area. Once mechanical allodynia is resolved, following the predefined objective criteria (detailed later in the methods), phase 2 focuses on assessing and treating tactile sensibility in the previously affected mechanical allodynia area. Phase 2 assessments include the pressure perception threshold, static two-point discrimination test, and vibration perception threshold. SR recommends continuing intervention by treating tactile sensibility in the area newly freed from mechanical allodynia with direct stimulation to facilitate optimal recovery, help reduce unprovoked pain, and reduce the risk of allodynia recurrence.

The procedure for managing and monitoring patients at the Center adhered to the standardized SR method. Patients maintained a weekly appointment at the Center, in addition to prescribed daily exercises to be performed at home. Each patient received treatment from two occupational therapists, who alternated on a weekly basis, and had received standardized, manualized training in SR evaluation procedures and intervention, before participant enrollment. Standardized assessment results were recorded by all therapists in the database. The therapists (pairs) were not consistently the same for each patient. A brief description of the standardized assessments and intervention procedures is provided in the following sections. For more in-depth information, refer to the SR handbook and a knowledge transfer article, which offer detailed descriptions of these assessments and intervention procedures [9,20].

The *Questionnaire de la douleur St-Antoine* (QDSA) is a French pain questionnaire designed to assess pain quality. Similar to the McGill Pain Questionnaire, it evaluates pain quality, albeit with a distinct structure and classification of pain qualifiers. The QDSA comprises 58 items (pain qualifiers), categorizing them into nine sensory classes and seven affective classes. Patients selected one item per class, and this chosen item was scored on a five-point Likert scale (ranging from 0 for no pain to 4 for very strong pain). Subscores were calculated for the sensory subscale (36 points) and the affective emotional subscale (28 points), with a possible total score of 64 points [18,19]. A higher score indicated a more intense qualification of pain. In both previous SR studies [11] and in this present study, the QSDA was employed to characterize provoked and unprovoked pain. The questionnaire was administered for the first time during the initial session with the therapist or subsequent sessions if time constraints arose. Subsequent assessments were conducted approximately monthly after the completion of the initial QDSA, spanning the entire duration of SR intervention, encompassing phases 1 and 2.

Phase 1: assessments for mechanical allodynia

Phase 1 of SR involved assessments and interventions targeting mechanical allodynia. Initially, the presence of mechanical allodynia was determined using an objective criterion based on the application of a 15.0-g Semmes-Weinstein monofilament to the perineum region, including the vaginal entry. The test was considered positive for mechanical allodynia when pain was triggered or exacerbated, indicating induced or increased pain beyond predetermined thresholds upon contact with the 15.0-g monofilament. The first step in the testing process was to assess the baseline severity of pain experienced by the patient at rest, representing the intensity of unprovoked pain. This assessment utilized VAS, which evaluated the actual intensity of pain at rest on a 100 mm line, ranging from no pain (0) to the worst pain imaginable (100). It should be noted that patients with mixed pain, as included in this study, experienced unprovoked pain at rest alongside touch-provoked pain. According to SR criteria, patients with either provoked or mixed pain should register a score of 30 mm or more on the VAS scale when touched by a 15.0-g monofilament to be considered for mechanical allodynia treatment. In cases of mixed pain, where pain at rest equals or exceeds 20 mm, an increase of at least 10 mm compared to the resting pain level was required when touched with the 15.0-g monofilament for mechanical allodynia to be considered for treatment (i.e., if pain at rest was 40 mm, it must increase to 50 mm with the 15.0-g monofilament) [9].

Mechanical Allodynia Assessments

Once the presence of mechanical allodynia was confirmed based on the criterion described above, additional assessments were employed to further characterize and refine interventions. In Phase 1, two additional mechanical allodynia assessments were conducted: allodynography, aimed at identifying the skin area exhibiting mechanical allodynia, and the Rainbow Pain Scale, used to identify the severity of mechanical allodynia.

**Figure 2:**
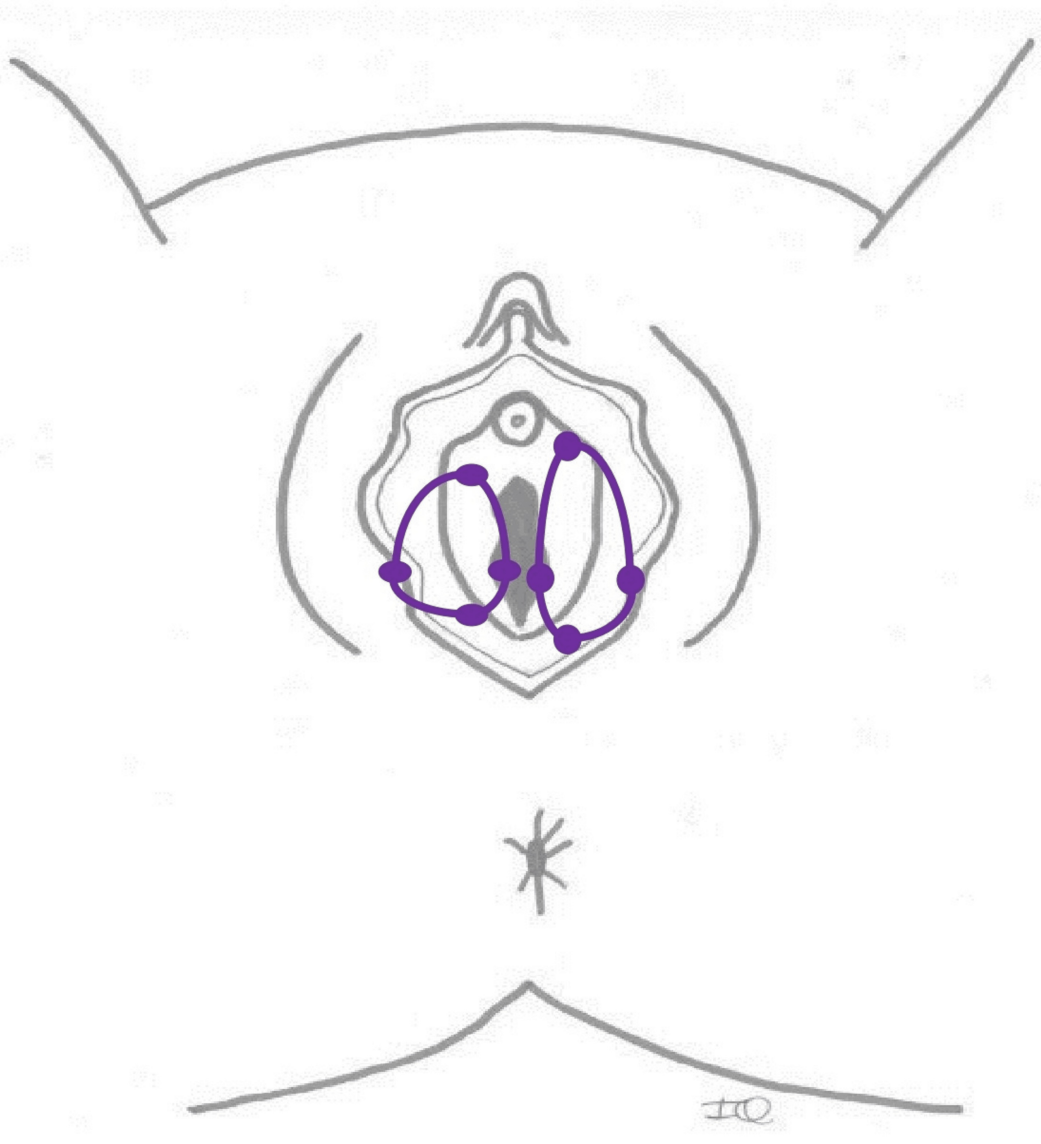
Example of an allodynography of the pudendal nerves (bilateral) on the perineum. The boundaries of the allodynic area are determined along two axes in relation to the vaginal entry: mediolateral and anteroposterior. Original figure created by the first author (IQ).

**Figure 3:**
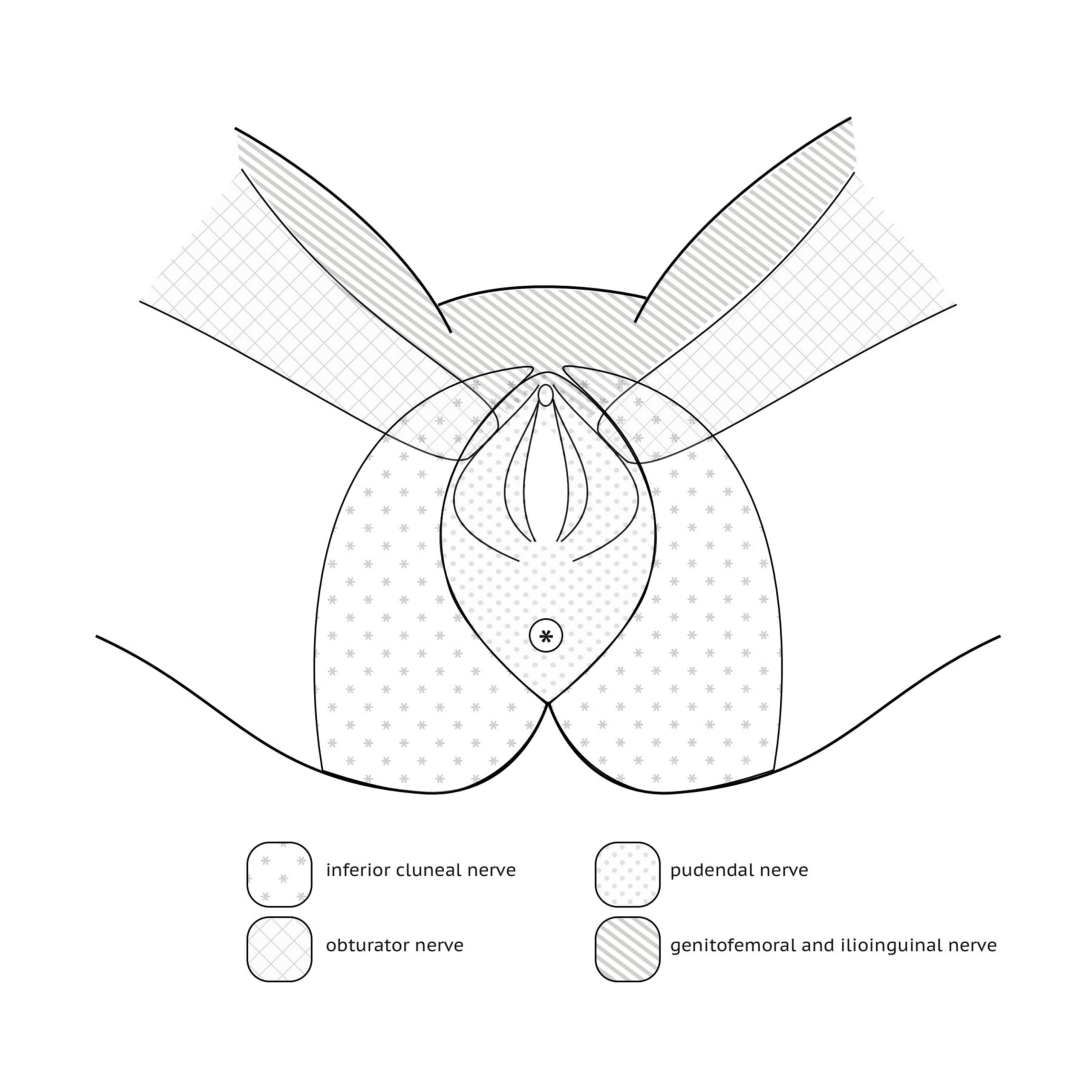
Cutaneous region of the external female genitalia nerves, including the pudendal nerve. Original figure created by the first author (IQ).

Allodynography: This assessment, illustrated in Figure 2, aimed to delineate the skin area exhibiting mechanical allodynia triggered by the touch with a 15.0-g Semmes-Weinstein monofilament applied for two seconds (interstimulus interval: eight seconds). Allodynography was used to identify the boundaries, i.e., the outermost points, of a cutaneous area where mechanical allodynia can be elicited with a 15.0-g force. These boundaries were determined along the mediolateral and anteroposterior axes in relation to the center of the region innervated by a specific nerve corresponding to the area with allodynia, specifically the pudendal nerve for this study [21,22]. The cutaneous region of the pudendal nerve is shown in Figure 3. The skin surface with mechanical allodynia (cm^2^) could then be estimated by multiplying the height (anteroposterior) and the width (vaginal entry-lateral border) of the allodynography. This assessment was conducted by the therapist during the first in-clinic appointment and subsequently every week or two weeks throughout Phase 1 of SR.

Rainbow Pain Scale: This assessment aimed to determine the severity of mechanical allodynia. A series of seven Semmes-Weinstein monofilaments, color-coded to represent mechanical allodynia severity, was used: 0.03 g (red), 0.2 g (orange), 0.7 g (yellow), 1.5 g (green), 3.6 g (blue), 8.7 g (indigo), 15.0 g (violet) [9,17]. They were systematically applied for two seconds each, with an eight-second interstimulus interval, in ascending order, from the smallest to the largest, to the center of the previously identified skin area with allodynia. Severity was determined by the first monofilament in the series that caused pain exacerbation, as described earlier. This assessment was conducted by the therapist during the initial in-clinic appointment and subsequently on a weekly basis throughout Phase 1 of SR.

Mechanical Allodynia Interventions

Phase 1 encompassed two daily intervention modalities: the application of precautions by the patient and distant vibrotactile counter stimulation.

Precautions: During weekly visits to the Center, patients received education on precautions to avoid touching the skin area with mechanical allodynia in their daily activities, such as getting dressed and bathing. They were instructed on strategies to adapt their activities accordingly. Examples of activity adaptation included wearing soft underwear and reducing or avoiding sexual intercourse.

Distant vibrotactile counter stimulation: In-clinic visits involved the application of distant vibrotactile counter stimulation by the therapist, utilizing therapeutic vibratory mechanical stimulation using Vibradol™ (frequency: 100 Hz, amplitude: 0.06 mm) [9]. This stimulation was applied once per visit, lasting for one minute, at a distance from the area with mechanical allodynia with an ipsilateral territory predetermined by the therapist. This was a pain-free proximal territory related to the pudendal nerve (e.g., ilioinguinal nerve), thoracic nerve 12, or a more proximal cutaneous territory (e.g., thoracic nerve 11 or 10) if the distal options were uncomfortable. The home exercise program involved distant tactile counter stimulation using a soft, comfortable fabric (e.g., silk, fur) in the same pain-free territory determined during the therapist’s application of Vibradol™. Women were encouraged to perform these exercises eight times per day for one minute, or less than one minute if the stimulation became uncomfortable or painful.

Phase 2: assessments and interventions for tactile sensibility

Phase 2 was initiated upon the resolution of mechanical allodynia, as determined by the 15.0-g criterion described previously [9].

Tactile Sensibility Assessments

Phase 2 began with assessments aimed at characterizing tactile sensibility to monitor its progression during this phase of SR. It included three assessments of tactile sensibility, using the pressure perception threshold, static two-point discrimination, and vibration perception threshold tests.

Pressure perception threshold: Semmes-Weinstein monofilaments were employed to establish the smallest static force (in g) detectable by the patient on the mechanical allodynia area identified in Phase 1 [9]. A lower pressure perception threshold result indicates better tactile perception.

Static two-point discrimination test: A two-point aesthesiometer was used to determine the minimum distance between two points at which patients could distinguish one or two static touches [9,12]. A shorter distance denotes better tactile discrimination.

Vibration perception threshold: The Vibradol™ generator, featuring a fixed vibration frequency of 100 Hz and variable amplitude (between 0.00 mm and 1.00 mm) [9], was used to determine the minimum amplitude of mechanical vibration detectable by the patient at the center of the allodynic skin area identified in Phase 1. A smaller vibration perception threshold result indicates better vibration perception. This result served as a guide for determining the amplitude of vibration used during therapy sessions (described below).

Interventions for Tactile Sensibility

Phase 2 involved the following two intervention modalities: in-clinic direct vibratory stimulation and home-based direct tactile stimulation with fabrics.

Direct vibratory stimulation: During weekly visits at the Center, therapeutic direct vibratory stimulation was administered using Vibradol™ (frequency: 100 Hz) with an amplitude of 0.1 mm above the previously determined vibration perception threshold [9]. This stimulation, lasting for five minutes, was directly applied to the prior allodynic skin area.

Direct tactile stimulation with fabrics: The home-based exercises entailed direct tactile stimulation of the prior allodynic skin area with three different fabrics (e.g., microfiber towel, sponge, paintbrush), alternating fabrics for each exercise period [9]. At regular intervals of a few seconds, patients were invited to touch an area of the skin with normal tactile sensibility adjacent to the affected area, facilitating a comparison in sensitive perception between the two areas. These exercises were progressed over the course of the program: from 12 times per day for 15 seconds during the first week following mechanical allodynia resolution, to four times per day for five minutes from the fifth week onwards [9]. Additionally, over the weeks of intervention for improving tactile sensibility, patients were prompted to use progressively less coarse fabrics for stimulation.

Analysis

Sociodemographic characteristics were described for each patient. Baseline, intermediate, and final data for pain quality, mechanical allodynia, and tactile sensibility assessments were individually detailed for each patient. Descriptive statistics, including median (med), minimum and maximum scores (range), and interquartile ranges (IQRs), were used to capture variations in individual pain quality, mechanical allodynia, and tactile sensibility outcome measures for each patient.

## Results

Patient characteristics

**Figure 4:**
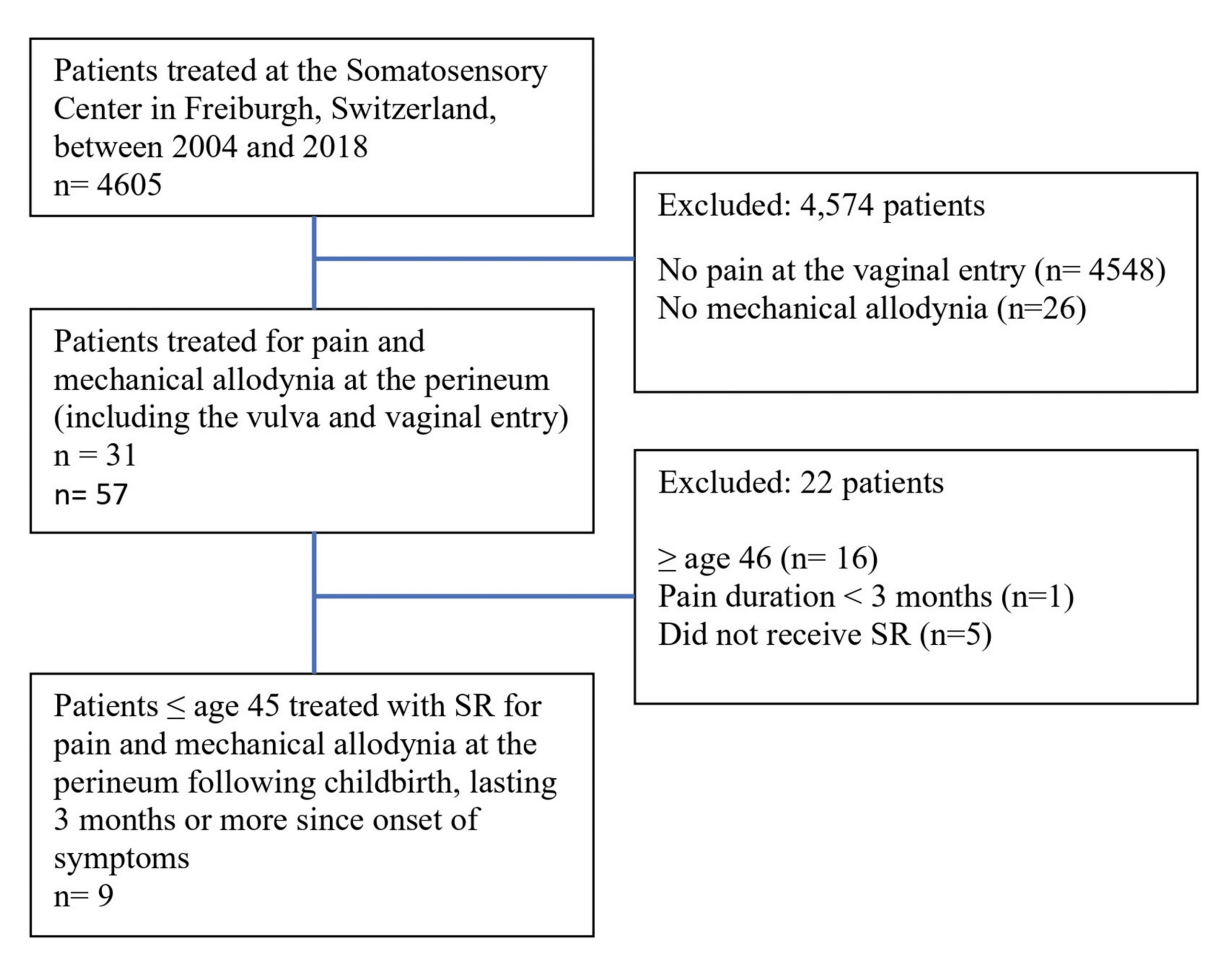
Study flow diagram for patient inclusion from the Somatosensory Rehabilitation Center (Freiburgh, Switzerland) database. SR: somatosensory rehabilitation

Figure 4 shows the selection process for patient inclusion. Nine women between the ages of 25 to 36 years old (median = 32.7) with PPPP were included. None of the selected patients discontinued the interventions. Sociodemographic information for these women is summarized in Table 1. However, for one patient (P9) (11%), data on all sociodemographic characteristics were missing, and only partial data were available for pain quality and mechanical allodynia assessments. All patients (n = 9, 100%) were referred to the Center by either a gynecologist or a family physician.

**Table 1:** Sociodemographic data. *: Duration of symptoms before the time of the first consultation at the Somatosensory Rehabilitation Center. Y: yes; n-a: non-available information; Med: median; IQR: interquartile range

	Patients									Med (IQR)
	P1	P2	P3	P4	P5	P6	P7	P8	P9	
Age (years)	29	34	25	33	35	29	36	28	32	32 (28.5-34.5)
Partner	Y	Y	Y	Y	Y	Y	Y	Y	n-a	-
Parity (number)	1	2	1	2	2	1	1	1	n-a	1 (1-2)
Duration since symptom onset (months)*	17	100	9	30	9	12	13	13	5	13 (9-23.5)

During delivery, one (11%) patient experienced a first-degree natural tear (P6), two (22%) had a second-degree natural tear (P3, P5), four (44%) underwent episiotomy (P1, P2, P3, P4), and one (11%) had an instrumental delivery (P3). A history of vaginal tear during a previous labor was documented for one (11%) patient (P8). Medication use was recorded for one (11%) patient (P3) (Dafalgan 1 g QID, Ponstan 500 mg). Pre-SR interventions were documented for one (11%) patient (P7) (11%) (osteopathy). However, interventions performed outside the Center during SR were not documented for any of the women.

All women (n = 9, 100%) reported perineal pain, including pain at the vaginal entry, which falls within the sensory cutaneous region of the pudendal nerve. The median duration between the onset of the initial symptoms at the vaginal entry and the first consultation at the Somatosensory Rehabilitation Center was 13 months (range = 5-100, IQR = 9.0-23.5) (Table 2). Regarding pain presentation, five (56%) women reported only provoked pain (P1, P3, P4, P6, P8) while four reported mixed pain (P2, P5, P7, P9). Although not systematically investigated in every patient, eight of them initially self-reported pain during intercourse (n = 8, 89%), a reduced frequency of intercourse (n = 4, 44%), pain when wearing tampons or sanitary pads (n = 4, 44%), and difficulty wearing pants or underwear (n = 3, 33%).

**Table 2:** Individual treatment durations, mechanical allodynia, and tactile sensibility results for each patient. Data are presented both in individual and aggregated form; no inferential statistical analyses were performed. RPS: Rainbow Pain Scale; VAS: Visual Analog Scale; PPT: pressure perception threshold; STD: static two-point discrimination test; VPT: vibration perception threshold; Med: median; IQR: interquartile range; n-a: non-available information

		Patients									Med (IQR)
		P1	P2	P3	P4	P5	P6	P7	P8	P9	
Initial allodynic surface (cm^2^)		15	10	68	1	48	2	11	3	n-a	10.5 (2.25-39.75)
Initial RPS		Yellow (0.7 g)	Violet (15.0 g)	Violet (15.0 g)	Violet (15.0 g)	Violet (15.0 g)	Blue (3.6 g)	Violet (15.0 g)	Violet (15.0 g)	Violet (15.0 g)	15.0 (9.3-15.0)
Treatment duration needed for Phase I for allodynia abolition (days)		40	16	49	7	26	21	42	43	34	34.0 (18.5-42.5)
Treatment duration for Phases I and II, for the entire intervention (days)		85	123	74	63	41	77	91	76	77	77 (68.5-88.0)
PPT (g)	Initial	0.6	0.6	0.6	0.3	0.6	0.2	1.0	0.6	0.5	0.6 (0.4-0.6)
	Final	0.1	0.2	0.4	0.04	0.2	0.05	0.04	0.2	0.1	0.1 (0.05-0.2)
STD (mm)	Initial	56	10	30	18	30	35	48	48	25	30 (21.5-48.0)
	Final	21	5	25	10	15	15	18	25	12	15 (11.0-23.0)
VPT (amplitude in mm)	Initial	0.06	0.04	0.06	0.05	0.05	0.07	0.14	0.05	0.09	0.06 (0.05-0.08)
	Final	0.06	0.03	0.06	0.05	0.04	0.03	0.04	0.03	0.06	0.04 (0.03-0.06)

Clinical characteristics at baseline

As for the initial QDSA, from which we obtained data from eight (89%) women, a broad range of pain qualifiers was reported by patients (32 out of 58 possible qualifiers). Data on QDSA are missing for one woman (11%), as it was impossible to complete the questionnaire due to her mother tongue not being French. The most frequent QDSA qualifiers reported initially, chosen by at least three women to describe their pain, were “burning” (n = 5), “tugging” (n = 5), “stinging” (n = 4), “numbness” (n = 4), and “itching” (n = 3). The most recurrent affective qualifiers of the QDSA were “fearful” (n = 5), “terrifying” (n = 4), “unbearable” (n = 4), “annoying” (n = 4), and “troublesome” (n = 3). At baseline, considering all included women, the median area of allodynography covering the perineum, including at least the vaginal entry, was 10.5 cm^2^ (range = 1-68, IQR = 2.25-39.75). For the Rainbow Pain Scale baseline assessment, the monofilament eliciting exacerbation of pain was violet (15.0 g) for seven women (78%), and blue (3.6 g) and yellow (0.7 g) for the two other patients (22%).

Changes in clinical measures of pain quality, mechanical allodynia, and tactile sensibility

**Figure 5:**
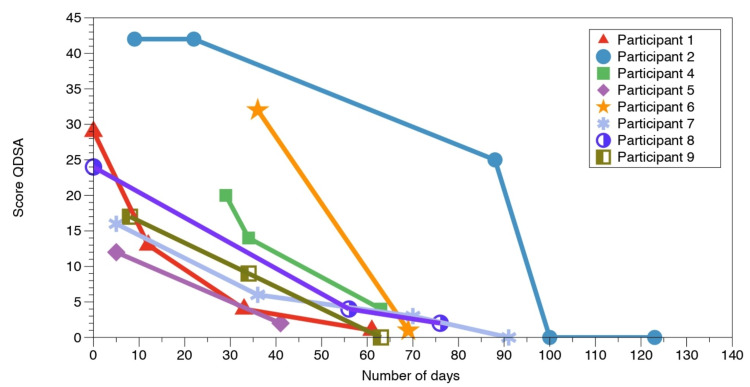
Individual QDSA scores throughout the entire somatosensory rehabilitation intervention (Phases 1 and 2). QDSA: *Questionnaire de la douleur Saint-Antoine*

The initial and final outcomes for pain quality, mechanical allodynia, and tactile sensibility measures, along with the treatment durations, are detailed in Table 2. When considering changes in pain quality measurements occurring over the entire duration of SR (i.e., combining Phases 1 and 2), all patients (n = 9, 100%) reported a less intense qualification of pain, with a median decrease of 20 points (range = 7-42, IQR = 16-30) on the QDSA. Intermediate QDSA scores (i.e., between initial and final values) were available for eight women, so the detailed time course of individual pain changes over the course of SR is presented in Figure 5.

All nine (100%) women achieved complete resolution of mechanical allodynia, as defined by the criterion of no significant exacerbation of pain upon touch with a 15.0-g Semmes-Weinstein monofilament, and as assessed through both allodynography and the Rainbow Pain Scale. The median duration of the SR intervention required in Phase 1 to resolve mechanical allodynia according to this criterion was 34 days (range = 7-49, IQR = 18.5-42.5). The median total duration of Phases 1 and 2 combined (i.e., duration of interventions to resolve allodynia and treat tactile sensibility) was 77 days (range = 41-123, IQR = 68.5-88.0), involving a median of 10 in-clinic interventions (range = 5-17, IQR = 9.5-12.5).

Once the criterion for pain exacerbation with a 15.0-g monofilament was no longer met, tactile sensibility was assessed in the same skin area as the previous mechanical allodynia (i.e., vaginal entry) in all women. It is noteworthy that during Phase 2, measures of tactile sensibility improved in all women (n = 9, 100%). The median change in pressure perception threshold from Phase 2 baseline to the end of SR was a decrease of 0.4 g (range = 0.15-0.96, IQR = 0.23-0.45). Improvements were also observed in the static two-point discrimination test, with a median decrease of 15 mm (range = 5-35, IQR = 6.5-26.5). The results of the vibration perception threshold test showed a slight reduction of 0.01 mm in median amplitude (range = 0.00-0.10, IQR = 0.00-0.035).

## Discussion

This retrospective case series study is the first to explore the use of SR to treat patients with PPPP and describe changes in pain and tactile sensibility in this population. Additionally, it demonstrates positive individual outcomes in women with PPPP undergoing SR. Indeed, according to a 15.0-g monofilament pain exacerbation criterion, mechanical allodynia was resolved in all patients treated with SR. Furthermore, individual scores on the QDSA decreased in all patients during SR interventions, indicating that patients perceived pain as qualitatively less intense. Following the resolution of mechanical allodynia, characterized by the pain exacerbation criterion, we hypothesize that underlying tactile hypoesthesia was revealed in the previously allodynic region in all patients. Although normative data for these outcome measures in the general population are lacking, one study with a small sample size (n = 30) estimated that the average detection threshold for light touch at the vaginal entry, assessed using Semmes-Weinstein monofilaments, is approximately 0.02 g [23]. In the present study, as patients had individual initial pressure perception threshold scores higher than 0.02 g (range = 0.2-1.0 g) after abolition of allodynia, this supports the hypothesis of tactile hypoesthesia present at the site of the former allodynia. Furthermore, due to observed improvements in all patients for the pressure perception threshold and static two-point discrimination tests during Phase 2 of SR in this study, we can speculate that the initial tactile sensibility results following the resolution of mechanical allodynia could be further enhanced, possibly because they were initially outside the normal range. The vibration perception threshold test was the only outcome that did not change over time.

The mechanisms potentially contributing to the beneficial effects of SR, as suggested in this study and previous ones [10-13], remain unknown. SR principles are based on the hypothesis that it stimulates both the peripheral and central nervous systems, inducing changes via neuroplasticity mechanisms, ultimately contributing to enduring pain reduction [21]. While many factors may be involved in PPPP, one of the primary factors could have a mechanical origin. In some cases, the pudendal nerve may sustain damage during delivery due to its anatomical location [24]. Consequently, PPPP could be partially attributable to a peripheral nerve injury. Such nerve lesions could also lead to symptoms associated with neuropathic pain, on which SR may purportedly have an effect through nervous system stimulation. In this context, it is possible that patients in this case series experienced neuropathic pain. Indeed, the most frequently chosen QDSA qualifiers by patients, such as numbness, burning, and itching, as well as the presence of mechanical allodynia, are associated with neuropathic pain, as described in the items of questionnaires used to screen for this type of pain (i.e., Douleur Neuropathique in 4 Questions, Leeds Assessment of Neuropathic Symptoms and Signs) [25,26].

Nevertheless, due to the multifactorial etiology of PPPP, we cannot exclude other factors that might have contributed to the positive outcomes observed in these patients. For instance, regarding breastfeeding as a factor implicated in PPPP [3], it is possible that the participation in SR coincided, for some women, with the cessation of breastfeeding, which could have contributed to a decrease in perineal pain. Another factor that cannot be ruled out is the effect of time, particularly in the natural recovery of this condition after childbirth. In addition, possible concurrent interventions and medications, undocumented for most patients, and the therapeutic relationship, could have influenced the results.

Interestingly, this is the first study to examine the presence, severity, and surface area of mechanical allodynia in women with PPPP using the Semmes-Weinstein monofilaments. Assessments involved the presence/absence of pain with the 15.0-g monofilaments, the Rainbow Pain Scale, and allodynography. The novel data from this study shed light on a potential underlying tactile hypoesthesia revealed by the resolution of mechanical allodynia in PPPP patients. This finding aligns with those from other studies in various non-genital persistent pain conditions, indicating that tactile hypoesthesia may manifest in the area of previously resolved mechanical allodynia [10,12]. Consequently, the identification and treatment of hypoesthesia becomes paramount. Moreover, this finding holds promise for advancing our understanding of the pathophysiological mechanisms implicated in the PPPP condition.

The study’s strengths lie in the execution of assessments, interventions, and data entry by therapists who received standardized, specialized training in SR. This approach ensured the uniform administration of SR across patients, thereby enhancing the internal validity of this study through protocol fidelity, consistent clinical assessments, and reliable data collection. Additionally, it should be noted that all patients consistently underwent this intervention for several weeks without treatment discontinuation. Furthermore, this study provides a sufficiently detailed description of the application of SR and its adaptation based on the evolution of the patient’s condition, rendering it easily replicable in clinical or research settings.

However, certain limitations should be acknowledged. First, the study’s design is retrospective, which may introduce inherent biases. Additionally, the small sample size and absence of a control group limit the generalizability of the findings. The lack of inferential statistics further restricts the strength of the study’s conclusions. Another limitation pertains to the use of non-validated outcome measures for the PPPP population. While the allodynography and the Rainbow Pain Scale have been standardized [9] and their reliability studied in painful hand conditions (i.e., complex regional pain syndrome, peripheral nerve injury, and recent hand fracture) [16,17], their validity in the context of PPPP remains unexplored. It is still unclear how these measures correlate with the patient’s symptomatology (e.g., pain intensity experienced during intercourse). Similarly, tactile sensibility tests (i.e., pressure perception threshold, static two-point discrimination test, vibration perception threshold) have been standardized [9], but lack validity and reliability measures in PPPP. Moreover, there are no normative values for these variables for these anatomic regions. The use of the QDSA introduces uncertainty regarding whether patients completed it in reference to provoked or unprovoked pain. Distinguishing between changes in these two types of pain and their associated circumstances (e.g., pain during intercourse) would have provided more nuanced insights. Moreover, the QDSA was not assessed during the initial in-clinic appointment for every patient. Consequently, it is conceivable that, for some patients, the initial score might have been higher if assessed from the outset, potentially leading to greater observed improvement. Another limitation pertains to the absence of patient-reported outcome measurements, except for the QDSA. This deviation from recommendations in the literature, which advocate for more patient-centered and meaningful outcome measures, is noteworthy [27]. Outcome measures in this study primarily focused on pain and sensory disturbances, making it challenging to establish relationships between these outcomes and sexual functioning. To address this limitation, it would have been relevant to incorporate more patient-rated outcome measurements, such as the Female Sexual Function Index [28] and the 36-Item Short Form Health Survey for quality of life [29]. Finally, home exercise adherence was not systematically tracked. Future prospective trials should incorporate standardized monitoring (e.g., logbooks or digital tracking) to examine dose-response effects.

Regarding intervention limitations, the duration remains approximate, as there was no precise criterion for stopping the intervention at the end of Phase 2, which therefore influenced this variable. Future studies should employ precise criteria to determine the cessation of interventions targeting tactile sensibility. A relevant endpoint could have been the restoration of normal tactile sensibility at the perineum, although norms for such restoration are currently unknown to our knowledge. Additionally, despite the absence of treatment discontinuation indicating patient adherence to SR, questions persist about the feasibility and acceptability of SR. For example, one may question whether the number of in-clinic interventions or the duration of the SR follow-ups is feasible and acceptable for patients. This consideration could prompt inquiries about the acceptability for patients to bear the cost of SR follow-ups or the acceptability of precautions to decrease contact with the perineum.

## Conclusions

This retrospective case series suggests that SR intervention could help reduce pain quality, mechanical allodynia, and improve tactile sensibility in patients with PPPP. Given that the methodological design did not allow us to assess efficacy, it can be concluded that SR merits further research to explore its use and effects as a potential therapeutic approach for women with PPPP. Therefore, recommendations for future research include characterization of pain thresholds, tactile sensibility, dyspareunia, and the use of patient-rated outcome measures (e.g., sexual function, quality of life) in the PPPP population undergoing SR. Moreover, other factors that may explain the pain experienced by PPPP patients should be documented (e.g., severity of perineal injury during delivery, previous and concomitant interventions, medication). In addition, future research in this field should incorporate aspects of feasibility and acceptability. Finally, a larger-scale randomized controlled trial with a greater sample size would enable us to investigate the efficacy of SR interventions in patients with PPPP.
